# Unravelling the Interplay between Cardiac Metabolism and Heart Regeneration

**DOI:** 10.3390/ijms241210300

**Published:** 2023-06-18

**Authors:** Fan Yu, Shuo Cong, En Ping Yap, Derek J. Hausenloy, Chrishan J. Ramachandra

**Affiliations:** 1 National Heart Research Institute Singapore, National Heart Centre Singapore, Singapore 169609, Singapore; 2 Cardiovascular & Metabolic Disorders Program, Duke-National University of Singapore Medical School, Singapore 169857, Singapore; 3 Yong Loo Lin School of Medicine, National University of Singapore, Singapore 119077, Singapore; 4 The Hatter Cardiovascular Institute, University College London, London WC1E 6HX, UK

**Keywords:** cardiac metabolism, ischemic heart disease, heart failure, heart regeneration, cardiomyocyte proliferation, angiogenesis, cardiac progenitor cells, mitochondria, reactive oxygen species, hormones

## Abstract

Ischemic heart disease (IHD) is the leading cause of heart failure (HF) and is a significant cause of morbidity and mortality globally. An ischemic event induces cardiomyocyte death, and the ability for the adult heart to repair itself is challenged by the limited proliferative capacity of resident cardiomyocytes. Intriguingly, changes in metabolic substrate utilisation at birth coincide with the terminal differentiation and reduced proliferation of cardiomyocytes, which argues for a role of cardiac metabolism in heart regeneration. As such, strategies aimed at modulating this metabolism-proliferation axis could, in theory, promote heart regeneration in the setting of IHD. However, the lack of mechanistic understanding of these cellular processes has made it challenging to develop therapeutic modalities that can effectively promote regeneration. Here, we review the role of metabolic substrates and mitochondria in heart regeneration, and discuss potential targets aimed at promoting cardiomyocyte cell cycle re-entry. While advances in cardiovascular therapies have reduced IHD-related deaths, this has resulted in a substantial increase in HF cases. A comprehensive understanding of the interplay between cardiac metabolism and heart regeneration could facilitate the discovery of novel therapeutic targets to repair the damaged heart and reduce risk of HF in patients with IHD.

## 1. Introduction

Ischemic heart disease (IHD) is the most prevalent cardiovascular disease and remains the leading cause of death globally. Although IHD-related mortality rates have decreased over time due to primary prevention, and improved diagnosis and treatment, the rise in absolute numbers of IHD cases is a serious cause for concern. Crucially, IHD can increase the risk of heart failure (HF) by 8-fold and, as expected, is the most frequent underlying cause of HF [1,2]. HF itself affects ~64 million individuals and is a leading cause of hospitalisation [3]. Currently, HF affects 1–1.3% of the global population and is expected to rise to 3% and affect more than 70% of those aged above 65 by 2030 [4]. While the ageing of the population will undoubtedly contribute to this foreseeable increase in HF prevalence, improved survival outcomes in IHD patients with acute myocardial infarction (AMI) may also contribute to an increase in HF cases [4,5]. As such, there is an unmet need to identify novel therapeutic strategies to prevent the onset and progression of HF in patients with IHD.

Myocardial ischemia instigates profound derangement in cellular energetics and metabolism in the heart which induces injury and eventual death of cardiomyocytes [6,7]. Cardiomyocytes undergo apoptosis at a rate of 0.002% in normal human hearts, 0.12–0.7% in failing hearts from patients with NYHA class III and IV [8,9,10], and a staggering 17% in ischemic hearts [10,11]. The adult human heart is one of the least regenerative organs, but it is widely accepted that the heart’s regenerative ability is preserved in mammals during the early neonatal stage, and throughout the entire lifespan in certain lower vertebrate species [12,13,14]. In support, cardiomyocyte proliferation has been observed in mice subjected to apical resection and MI within the first week of birth [14,15], but this regenerative window was restricted to 2 days postpartum in larger mammals [16,17]. Heart regeneration has also been observed in a human newborn with severe MI where functional cardiac recovery had occurred within weeks after the initial extensive myocardial damage, translating into long-term normal cardiac function [18]. While the ability to promote heart regeneration in the setting of AMI is appealing, very little is known about the molecular pathways underlying cardiomyocyte proliferation. Alternatively, several efforts have been made to transplant various cell types into the infarcted heart. However, suboptimal delivery, homing, engraftment, and survival of these transplanted cells remain a major issue for it to be considered as a viable therapeutic modality [19]. Interestingly, marked changes in cardiac metabolism have been found to occur during heart development as evidenced by the predominant utilisation of glycolysis in foetal hearts and oxidative phosphorylation in adult hearts, the latter coinciding with terminal differentiation and reduced proliferation of cardiomyocytes [20,21]. These findings lend support to the assumption that the transition from glycolytic to oxidative metabolism, and the resultant increase in reactive oxygen species (ROS) production is a key driver of DNA damage and cell cycle arrest in cardiomyocytes [22].

With the aim of understanding the complexities associated with cardiac metabolism and heart regeneration, we review the role of metabolic substrates and mitochondria in heart regeneration, and discuss potential targets aimed at promoting cardiomyocyte cell cycle re-entry.

## 2. The Role of Metabolic Substrates in Heart Regeneration

The heart beats around 100,000 times each day and consumes ~8% of total ATP produced by the body [11,23]. Despite being one of the most energy-consuming organs, the heart stores limited amounts of ATP, which are sufficient to maintain its function for only a few seconds in the presence of nutrient shortage [24]. In order to meet the high energy demands, the heart can utilise a variety of energy substrates (e.g., fatty acids, glucose, ketones, and amino acids), albeit at different proportions. Importantly, the heart can readily switch to a specific type of fuel during cardiac development, and in response to physiological and pathological stress [25]. In this section, we focus on the major metabolic substrates utilised by the heart and discuss their potential roles in heart regeneration.

### 2.1. Glucose Metabolism

Cells with high proliferative capacity display elevated glycolic rates for energy production, despite the presence of adequate oxygen [26]. Glycolysis is an inefficient way to generate energy as only 2 ATPs are generated per molecule of glucose (in contrast to 36 ATPs generated by oxidative phosphorylation); however, this metabolic pathway is a major contributor for the biosynthesis of cellular components, such as lipids, amino acids, and nucleotides [26]. Studies in zebrafish, and in neonatal and adult mice have shown a pro-glycolytic metabolic profile is favourable for heart regeneration. In support, analysis of the transcriptome and proteome profile in zebrafish at 7 days post-cryoinjury revealed an alteration in cardiomyocyte metabolism from mitochondrial oxidative phosphorylation to glycolysis at the border and remote zones [27]. In other studies, lactate, an end product of glycolysis, promoted cell cycle progression in neonatal mouse cardiomyocytes and in human induced pluripotent stem-cell-derived cardiomyocytes (iPSC-CMs) by regulating the expression of genes involved in cell fate and proliferation [28].

**Figure 1:**
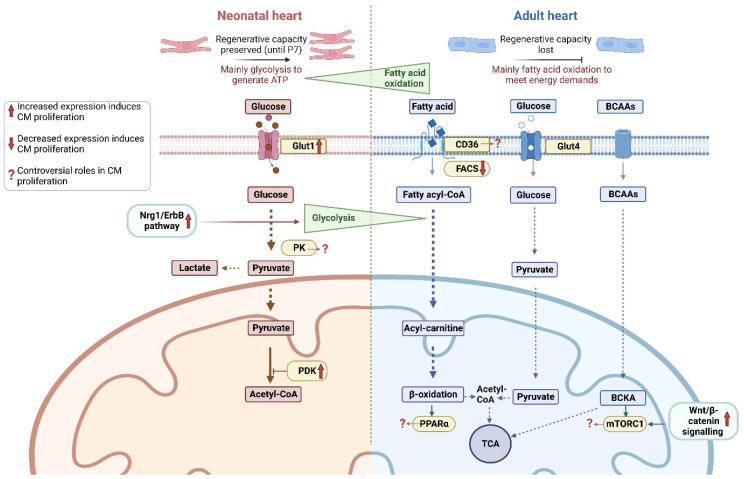
Schematic illustrating the interplay between cardiac metabolism and cardiomyocyte proliferation in neonatal and adult mammalian hearts. Neonatal heart metabolism (red), adult heart metabolisms (blue). Abbreviations: Glut1—glucose transporter type 1; Glut4—glucose transporter type 4; PK—pyruvate kinase; PDK—pyruvate dehydrogenase kinase; FACS—fatty acyl coA synthase; PPARα—peroxisome-proliferator-activated receptor alpha; BCAAs—branched-chain amino acids; BCKA—branch-chain alpha-keto acids; mTORC1—mammalian target of rapamycin complex 1; TCA—tricarboxylic acid cycle.

In neonatal and healthy adult hearts, glycolysis contributes to ~44% and ~5% of total ATP produced, respectively [29,30]. The cardiomyocyte plasma membrane is impermeable to glucose; as such, glucose uptake is mediated by glucose transporters (GLUTs) of which 14 members have been identified in various tissues to date. GLUT1 and GLUT4 are the most abundantly expressed isoforms in the heart [31], and notably, both transporters display an expression profile coinciding with different stages of cardiac development, with GLUT1 and GLUT4 being predominantly expressed in foetal and adult hearts, respectively [32]. The upregulation of GLUT1 has been found to play important roles in cardiac development and in response to cardiac stress [33,34]. Consistently, cardiac-specific overexpression of GLUT1 increased the percentage of proliferative cardiomyocytes and reduced fibrosis in cryoinjured neonatal mice by promoting nucleotide biosynthesis [35] (Figure 1). While these findings support the upregulation of GLUT1 as a potential mediator of heart regeneration, it is important to note that glycolysis is a fundamental metabolic pathway in active inflammatory cells (e.g., neutrophils, proinflammatory macrophages) [36], and inhibition of GLUT1 has been considered a potential therapeutic intervention for attenuating pro-inflammatory responses following AMI [37].

Besides their role in metabolism, certain glycolytic enzymes have been found to be interlinked with cell cycle regulatory pathways. Pyruvate kinase (PK) is an important enzyme which regulates the conversion of phosphoenolpyruvate and ADP to pyruvate and ATP [38]. These enzymes also show developmental-stage-specific expression profiles, with the PK muscle isoform 2 (PKM2) being predominantly expressed during embryonic and postnatal development, and PKM1 being the dominant isoform in adult cardiomyocytes [39]. Interestingly, cardiomyocyte-specific overexpression of PKM2 in post-MI adult mice has been shown to increase cardiomyocyte proliferation, and improved cardiac function and long-term survival by elevating targets downstream of β-catenin signalling [39]. Conversely, PKM2 expression has been found to be upregulated in stressed hearts where it has contributed to HF [40], while inhibition of the PKM2/β-catenin axis in post-MI mice reduced infarct size, increased the percentage of proliferative cardiomyocytes, improved mitochondrial function, and enhanced angiogenesis, which was attributed to the activation of target genes associated with cell proliferation [41]. Pyruvate dehydrogenase kinases (PDKs) are another family of enzymes with important roles in regulating pyruvate metabolism and metabolic flexibility [42]. Transcriptomic analyses in zebrafish revealed PDK enzymes, PDK2b, PDK3b, and PDK4b are up-regulated at the border zone in cryoinjured hearts, and overexpression of PDK3 increased the number of proliferating cardiomyocytes, albeit with no reduction in scared regions [43] (Figure 1).

Other signalling pathways not directly involved in glycolytic metabolism have also been implicated in cardiomyocyte proliferation. For instance, the Nrg1/ErbB pathway is indispensable for cardiac development as it regulates cardiomyocyte growth and survival and mediates terminal differentiation of cardiomyocytes from iPSCs [44,45]. The Nrg1/ErbB2 axis has also been shown to mediate profound changes in metabolism by up-regulating glycolytic genes at the border zone of cryoinjured zebrafish hearts [46] (Figure 1). Furthermore, cardiac-specific overexpression of ErbB2 after 3 weeks of permanent left anterior descending (LAD) coronary artery ligation in mice promoted de-differentiation and proliferation of cardiomyocytes, resulting in improved cardiac function [47]. While it is unclear how this Nrg1/ErbB2 axis mediates metabolic remodelling, it could be through the modulation of fatty acid metabolism as transient overexpression of ErbB2 in a breast cancer cell line promoted glycolysis and cell migration by upregulating fatty acid synthase involved in neoplastic lipogenesis [48,49].

Collectively, these findings suggest a switch from glucose oxidation to glycolysis is sufficient to promote cell cycle re-entry in adult cardiomyocytes (Figure 1). However, considering the ubiquitous expression of GLUTs in multiple cardiac cell types and that certain cell cycle activators are associated with cardiac hypertrophy and cancer [34,50,51], future strategies aimed at targeting these pathways should be investigated in a cell-type-specific manner to circumvent potential off-target effects.

### 2.2. Fatty Acid Metabolism

The foetal heart barely utilises fatty acids for efficient ATP production, which could be attributed to its low mitochondrial content and the limited availability of fatty acids in the placenta [52]. However, immediately after birth, the infant heart switches rapidly to fatty acid β-oxidation (FAO) [52,53], and this metabolic pathway generates the majority of ATP (~40–60% of total ATP) throughout adulthood [30]. Compared to other substrates, FAO generates the majority of ATP; however, as this process consumes the largest amount of oxygen, fatty acids are considered the least efficient energy substrate [54].

Long-chain fatty acid (LCFA) uptake into the cytosol is mediated by the fatty acid transport protein, CD36/FAT [54], and although the role of CD36 in cardiomyocyte proliferation is unclear, CD36 knockdown in mouse endothelial cells (ECs) has been found to prevent angiogenesis and vascular repair in response to hindlimb ischemia [55]. ECs play important roles in establishing an intact vasculature and in guiding cardiomyocyte organization in response to injury [56], while angiogenesis is essential for heart regeneration, as it restores blood flow to damaged myocardial tissue [57,58,59]. EC-specific inhibition of Notch signalling has been shown to impair fatty acid transport, resulting in abnormalities of the cardiac metabolome and vascular densities in adult mouse hearts [60]. In this study, genetic ablation of RBP-Jκ (a core component of the Notch pathway) reduced LCFA transport to the heart, resulting in a switch to glucose metabolism as the main source of energy, which, in turn, promoted cardiac hypertrophy and HF. Indeed, several studies support Notch signalling as a critical mediator of angiogenesis and cardiomyocyte proliferation [61,62], but it is unclear whether this regenerative ability is regulated by cardiac metabolism.

In the cytosol, LCFAs are esterified to fatty acyl CoAs by fatty acyl coA synthase and the resulting acyl groups are transferred into the mitochondria for FAO [54]. Acyl coA synthetase long-chain family member 1 (ACSL1) is a key rate-limiting enzyme which regulates LCFA uptake rates by increasing esterification to form fatty acyl CoA [63,64] (Figure 1). Interestingly, the expression of ACSL1 has been found to increase with age while the expression of glucose metabolism-related enzymes (glucose-6-phosphate 1-dehydrogenase, hexokinase 1, hexokinase 3, PKM) decrease with age [65]. These findings allow for the speculation that increased FAO during ageing may be an important suppressor of heart regeneration. In support, cardiac-specific knockdown of ACSL1 in neonatal mice promoted cardiomyocyte proliferation even at 60 days of age as evidenced by increased expression of cell proliferation markers [65]. Similarly, knockdown of ACSL1 in post-MI adult mice improved cardiac function by inducing cardiomyocyte proliferation. Mechanistic studies have revealed that inhibition of ACSL1 mediates cell proliferation via an Akt-FoxO1 axis, as suppression of this pathway decreased the expression of positive cell cycle regulators, cyclin B1, cyclin D2 and cyclin-dependent kinase 1 [65]. While these findings suggest a decrease in FAO is necessary for cardiomyocyte proliferation, other studies have shown that increased FAO (mediated by ACSL1) improves re-endothelisation after vessel injury. Furthermore, cyclic mechanical stretching of endothelial progenitor cells (EPCs) enhances vascular adhesion and endothelial differentiation by activating ACSL1 to increase FAO, while transplantation of ACSL1-overexpressing EPCs in rats with carotid injury results in improved vascular homing and repair [66].

Peroxisome-proliferator-activated receptors (PPARs) are key regulators of FAO [67]. PPARα is a master ligand-activated transcriptional factor that coordinates the expression of lipid metabolism genes, such as CD36 and mitochondrial FAO enzymes (e.g., carnitine palmitoyltransferases, acyl-CoA dehydrogenases) [68] (Figure 1). Experimental evidence suggests that PPARα plays a biphasic role in cell proliferation, as administration of the PPARα agonist, GW7647, was initially found to stimulate cardiomyocyte proliferation in infant mice at P4, but later promoted cardiomyocyte hypertrophy and binucleation, and reduced cardiomyocyte proliferation rates at P5 [69]. These findings suggest PPARα may have a more prominent role in the terminal differentiation of cardiomyocytes. Indeed, increased expression of PPARα has been observed during the differentiation of mouse embryonic stem cells into cardiomyocytes, while pharmacological inhibition of PPARα prevented this process, as evidenced by a decreased expression of cardiac specific genes [70].

Collectively, these findings support a role for FAO in promoting metabolic maturation in the adult heart with concurrent reduction in regenerative capacity [71,72]. It remains controversial whether inhibition of FAO is sufficient to extend the regenerative window as this may also delay cardiomyocyte maturation. Hence, the interplay between metabolic transitions and cardiomyocyte maturation needs to be carefully considered as inhibition of FAO at inopportune settings could result in adverse outcomes. In support, analysis of endomyocardial biopsies from failing hearts has revealed a decrease in *PPARA* mRNA, implying a decrease in FAO in the setting of HF [73]. Finally, recent findings suggest that neither GW7647 nor the FAO inhibitor, etomoxir, could promote cardiomyocyte proliferation in post-MI mouse hearts [74].

### 2.3. Ketone Body Metabolism

Ketone bodies such as acetoacetate, β-hydroxybutyrate, and acetone accumulate in the systemic circulation under conditions of prolonged fasting, insulin deprivation, and extreme exercise [75,76,77], with recent evidence supporting an increased utilisation of ketones in failing hearts [78]. The role of ketones in the developing heart is less clear. Comparative proteomics analysis of cells from three stages of cardiac differentiation (iPSCs, cardiac progenitor cells, and cardiomyocytes) has revealed ketogenic substrates are upregulated as result of increased expression of ketogenic enzymes, 3-hydroxymethyl-3-methylglutaryl-CoAlyase, 3-hydroxy-3-methylglutaryl-CoA synthase 2 (HMGCS2), and 3-hydroxybutyrate dehydrogenase 1 [79]. In another multi-omics study, increased ketogenesis was found to occur in neonatal mouse hearts at P7 when compared to hearts at E18.5 [80]. Interestingly, the expression of HMGCS2 (a rate-limiting enzyme of ketogenesis) gradually decreased after weaning and its levels reached normal physiological levels by postnatal day 56. Since the occurrence of early ketogenesis coincided with the regenerative window in neonatal mouse hearts, it could be speculated that ketone body utilisation plays a role in cardiomyocyte proliferation. In support of this idea, overexpression of HMGCS2 improved cardiac function in post-MI mice by increasing the percentage of phospho-histone H3 (PHH3)^+^ (a cell proliferation marker) cardiomyocytes [81]. Furthermore, ketone body oxidation was shown to enhance EC proliferation and angiogenesis in vitro and in mice subjected to pressure overload [82]. Whether this enhanced angiogenic capacity of ECs is sufficient to improve cardiac function remains to be validated as left ventricular (LV) ejection fraction was not investigated in this study, while the LV anterior wall thickness at end-diastole remained thickened even after ketogenic diet in the setting of pressure overload. As the primary concern for promoting ketone body oxidation is the risk of acidosis [83], future studies should investigate the safety range of circulating ketone bodies to maximise their potential benefits in heart regeneration, whilst preventing adverse effects.

### 2.4. Amino Acid Metabolism

Although amino acids are one of the smallest contributors of ATP production (~2% of total ATP) [84], they are intricately involved in several pathological conditions [30]. High circulating levels of the branched-chain amino acids (BCAAs), leucine, valine, and isoleucine, are associated with insulin resistance, type 2 diabetes, coronary artery diseases, and HF [85,86]. Interestingly, BCAAs were found to modulate liver regeneration and function in patients who had undergone hepatectomy [87]. BCAAs and their metabolites mediate cell growth, proliferation, and tumour progression by activating the mammalian target of rapamycin complex 1 (mTORC1) pathway [88] (Figure 1). Leucine, in particular, is a key stimulator of mTORC1-mediated cell growth through blockage of the mTORC1 inhibitor, sestrin 2 [89]. Given that mTORC1 is a mediator of cardiac hypertrophy [90], BCAAs may have an undefined role in cardiomyocyte proliferation and growth.

Multi-omics analysis has revealed that glutamine is enriched during heart regeneration in zebrafish and in neonatal mice but reduced in adult mice which have lost regenerative capacity [91]. This dynamic change in glutamine expression was found to correlate with the regulation of mTORC1, which plays an important role in cell growth and proliferation [92,93]. Interestingly, activation of the Wnt/β-catenin pathway in regenerating zebrafish hearts rescued the negative effects that mTORC1 inhibition exerted on cardiomyocyte proliferation [91] (Figure 1). Given that the Wnt/β-catenin pathway is a master regulator of cardiac development and cardiomyocyte terminal differentiation [94], further studies are needed to tease out the interplay between Wnt/β-catenin signalling and glutamine in cardiomyocyte proliferation [95].

In summary, the adult heart is incapable of repairing damaged tissue, which is, in part, attributed to the switch in metabolic substrate utilisation after birth. Accumulating evidence supports the possibility that multiple metabolic components are involved in the regulation of cardiomyocyte proliferation. Indeed, conditions of high glycolysis and reduced FAO have been proposed to promote heart regeneration, but the roles of ketone body and amino acid metabolism in cardiomyocyte proliferation is controversial and unclear. As such, future studies are needed to elucidate the dynamic interplay between these metabolic pathways and the cell proliferation pathways which they regulate.

## 3. The Role of Mitochondria in Heart Regeneration

The heart, a perpetually active organ with substantial energy needs relies on a dense and highly functional mitochondrial network to maintain energy requirements and overall performance [23,96]. However, the role of mitochondria in the heart extends beyond energy production, as these dynamic organelles also orchestrate a range of cellular functions, including signal transduction, calcium regulation, oxidative stress management, and apoptosis [97,98,99]. Recent findings have begun to shed light on the intricate link between mitochondria metabolism and cardiovascular diseases [100,101,102]. This includes understanding how early defects in mitochondrial oxidative phosphorylation manifest in HF, and the pivotal role of mitochondrial metabolic impairment in MI-induced cardiac damage [103,104,105].

The role of mitochondria has extended into the areas of cell fate determination and development [106]. Crucial functions of mitochondria in stem cells have been highlighted in several reports [107,108], indicating mitochondrial characteristics, such as morphology, localization, abundance, and function, could serve as markers of pluripotency, underscoring the multifaceted roles these organelles play in cardiac health and disease [106,109].

### 3.1. Oxidative Phosphorylation and Reactive Oxygen Species

**Figure 2:**
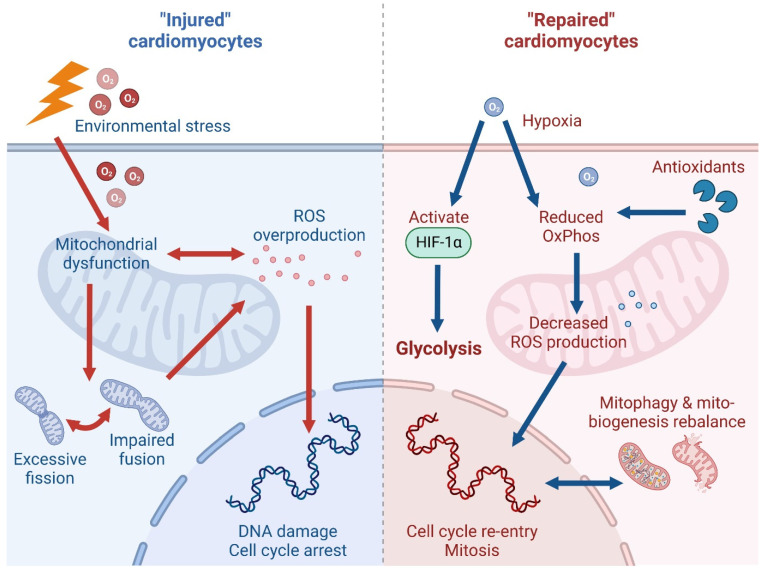
Schematic illustrating the role of mitochondria in cardiac injury and regeneration. (**Left**) environmental stress, such as that caused by ischemia/reperfusion injury can lead to mitochondrial dysfunction, resulting in ROS overproduction and dysregulation of mitochondrial dynamics, thereby instigating DNA damage and suppressing cardiomyocyte proliferation. (**Right**) under hypoxic conditions or during antioxidant treatments, cardiomyocytes can switch from oxidative metabolism to glycolysis, potentially via the HIF-1α pathway, and undergo restoration of aberrant mitophagy and mitochondria biogenesis to promote cell cycle re-entry. Abbreviations: ROS—reactive oxygen species; HIF-1α—hypoxia inducible factor 1α.

Mitochondria are the primary sites of oxidative phosphorylation, a process that generates ATP with superior efficiency compared to glycolysis [110]. However, this increased efficiency is linked with the endogenous generation of ROS as a by-product of ATP production due to electron leakage [111,112]. Overproduction of ROS can lead to oxidative DNA damage in cardiomyocytes, triggering a cell cycle checkpoint and arresting the cell cycle [22,113]. Notably, mitochondrial dysfunction, such as when induced by mitochondrial transcription factor A inactivation, has been shown to elevate ROS production, activate the DNA damage response, and induce cardiomyocyte cell cycle arrest, eventually leading to lethal cardiomyopathy [114]. Meanwhile, elevated ROS levels have also been linked to disturbances in mitochondrial and antioxidant proteins, leading to cardiac hypertrophy [115]. On the contrary, when using microRNA or CRISPR/Cas9 technology to silence key genes involved in the mitochondrial electron transport chain or tricarboxylic acid cycle, a reduction in mitochondrial number was observed followed by a decrease in ROS and an increase in cardiomyocyte proliferation [116,117]. These findings support the causal relationship between disturbed mitochondrial function and ROS production, which eventually interferes with cardiomyocyte proliferation (Figure 2).

In heart diseases such as AMI, redox (reduction–oxidation) changes in the injured heart may affect the proliferation and differentiation of cycling cardiomyocytes or progenitor cells [118,119]. This indicates that ROS levels generally correlate with stem cell differentiation, and increased oxidative stress post-cardiac injury could potentially induce the terminal differentiation of glycolytic cardiac progenitor cells (CPCs) [120,121]. Furthermore, cardiac hypoxia, a common consequence of cardiac injury, may play a crucial role in the recruitment of cycling cells or their progeny to the injured site [122]. Ineffectively managed ROS levels are also reported to be associated with cardiac ageing, cardiomyopathy, and a decline in the CPC population, resulting in reduced cardiac cell turnover [123,124].

Given the critical roles of mitochondrial function and ROS production in cardiac health, targeting these mitochondrial injuries with an emphasis on reducing oxidative damage could offer a promising strategy to delay the progression of HF.

### 3.2. Hypoxia Conditioning

The heart was initially recognised as the least regenerative organ, but this notion has been significantly developed over the past few decades [125,126]. Accumulating evidence suggests the existence of progenitor cells, such as c-kit^+^ cells, stem cell antigen-1^+^ cells, side population (SP) cells, and cardio-sphere-derived cells, within the adult heart, albeit in limited numbers [22,127,128,129]. These resident CPCs have demonstrated remarkable capabilities for self-renewal and differentiation into multiple cardiovascular lineages, including endothelial, smooth muscle, and myocardial cells, and this phenomenon is observable both in vitro and in vivo [19,130].

Studies have shown that a hypoxic niche environment may regulate signalling pathways to sustain the dedifferentiation and survival of foetal cardiovascular progenitor cells [131,132], whereas high oxygen concentrations coincide with the stagnation of cardiomyocyte proliferation [133]. Moderate hypoxia (SaO2 75–85%) can also bolster cell cycle activities in postnatal human cardiomyocytes [134]. Along these lines, the preserved self-renewal and decreased mitochondrial ROS levels were further observed in murine CPCs residing in hypoxic niches [135]. Moreover, intracellular ROS production was found to be maintained at low levels in several resident CPCs in the adult heart to preserve their quiescence and/or multipotency [119].

Long-term systemic hypoxemia could potentially reduce mitochondrial respiration and consequent ROS production in adult cardiomyocytes, which may promote cardiomyocyte re-entry into the cell cycle, thereby stimulating the proliferation of terminally differentiated cardiomyocytes [119,136] (Figure 2). Interestingly, exercise such as treadmill running have been shown to effectively reduce cardiomyocyte ROS accumulation and induce mitochondrial uncoupling, which coincided with heart regeneration [133]. Similarly, gradual exposure to severe systemic hypoxemia in mice resulted in the inhibition of oxidative metabolism, decreased ROS production and oxidative DNA damage, and reactivation of cardiomyocyte mitosis [137]. Besides ROS, other potential mechanisms through which hypoxia attenuates cardiac stem cell apoptosis have been identified. For instance, low oxygen tension has been shown to stabilize and activate several critical transcription factors and signalling pathways, such as hypoxia inducible factor 1α (HIF-1α) and Yes-associated protein (YAP), which, in turn, induced metabolic remodelling towards glycolysis to facilitate cardiomyocyte proliferation [138,139]. Importantly, HIF-1α and YAP control glycolysis by regulating the expression of glycolytic enzymes, and deletion or downregulation of these players has been shown to impair glucose uptake and glycolysis, which decreased cardiomyocyte proliferation [138].

Hypoxia-induced proliferation has been shown to be sufficient for promoting heart regeneration following MI [113]. Hypoxemia exposure following MI appears to induce robust regeneration, reduce myocardial fibrosis, and improve LV systolic function [137]. Additionally, a moderate level of hypoxia in combination with a mitochondrial ROS scavenger reversed the hypertrophic growth of LV cardiomyocytes by inducing cell cycle re-entry in terminally differentiated cardiomyocytes, thereby resulting in a substantial recovery of cardiac function [112].

The therapeutic potential of hypoxia/antioxidant treatment extends beyond resident CPCs. Hypoxia conditioning and antioxidant treatment can also benefit transplanted cardiac stem/progenitor cells aimed at repairing infarcted hearts, as this approach is challenged by low survival rates of donor cells [140,141]. For instance, hypoxic preconditioning of c-kit^+^ CPCs improves their survival and homing after engraftment into an infarcted heart [142]. Similarly, overexpressing sulfiredoxin 1, reduces ROS generation, and mitochondrial membrane potential, while enhancing the primary antioxidant systems and increasing the migration, proliferation, and cardiac differentiation of CPCs [143]. Another antioxidant, MHY-1684, has been shown to enhance the angiogenic capacity of CPCs in the setting of ROS-related diabetic cardiomyopathy by decreasing hyperglycemia-induced mitochondrial ROS generation and attenuating mitochondrial fragmentation. These findings indicate a potential therapeutic role for antioxidants in modulating mitochondrial dynamics and function in response to oxidative stress [142].

### 3.3. Mitochondrial Quality Control

Mitochondrial dynamics, encompassing biogenesis, fusion, fission, and mitophagy are crucial for regulating mitochondrial morphology and maintaining mitochondrial health, especially for cellular responses to metabolic cues or environmental stresses [144,145]. Abnormal mitochondrial dynamics such as excessive mitochondrial fragmentation and impaired fusion have been implicated as drivers of HF and cardiac ischemia/reperfusion (IRI) injury [146,147], while maintenance of normal mitochondrial structure and function could facilitate heart regeneration [148]. There is growing evidence suggesting that mitochondrial quality control in cardiomyocytes can enhance cardiac function, save cardiomyocytes from death, and prevent worsening of cardiovascular diseases under external environmental stress [144,149,150] (Figure 2).

Studies have shown that the differentiation process of c-kit^+^ progenitor cells involves mitochondrial fragmentation, which is controlled by the calcineurin-Drp1 pathway [151], while an environmental stressor such as high glucose milieu can alter mitochondrial dynamics and increase the expression of fission-related proteins such as Fis1 and Drp1, leading to a significant decrease in the tube-forming ability of CPCs [152]. Interestingly, pharmacological inhibition of mitochondrial fragmentation can help to maintain the undifferentiated state of c-kit^+^ progenitor cells. Mitochondrial division inhibitor 1 (mdivi-1), which inhibits Drp1-dependent mitochondrial fission, has shown promise in enhancing the survival of human W8B2^+^ cardiac stem cells, but surprisingly, the cytoprotective effects of mdivi-1 in simulated IRI models does not appear to be governed by changes in mitochondrial morphology, membrane potential, or ROS production [153].

The balance of mitophagy and mitochondrial biogenesis is of great importance in cardiomyocyte proliferation and differentiation. Mitophagy is induced during differentiation of adult CPCs and is mediated by mitophagy receptors [154]. Disrupting BNIP3L- and FUNDC1-mediated mitophagy during differentiation leads to sustained mitochondrial fission and the formation of dysfunctional mitochondria, resulting in increased susceptibility to cell death and failure to survive in an infarcted heart [155]. In a hypoxia/reperfusion injury cellular model, transfection of miR-494-3p mimic (inhibitor of PGC1α) improved cardiomyocyte proliferation activity by inhibiting mitochondrial biogenesis, thereby preventing the occurrence of cardiomyocyte apoptosis and autophagy [156].

In summary, the intricate role of mitochondria in cardiac regeneration is woven into multiple facets of cellular processes. Mitochondrial oxidative phosphorylation and ROS play pivotal roles in powering the energy-demanding process of cardiomyocyte proliferation, whereas mitochondrial dynamics and mitophagy are crucial in upholding mitochondrial quality control, thereby, mutually facilitating the regeneration of robust cardiac cells. Enhancing our comprehension of the mechanisms underlying mitochondrial dysfunction and identifying innovative ways to bolster mitochondrial function and foster heart regeneration could pave the way for novel therapeutic strategies.

## 4. Potential Strategies to Promote Heart Regeneration through Metabolic Modulation

In the past decade, studies have focused on cell-based therapies to promote heart regeneration by administrating stem cells into injured hearts [157]. Several clinical trials have been conducted which evaluated the direct intracoronary or intramyocardial delivery of multiple sources of adult stem cells (e.g., bone marrow cells and adipose tissue derived cells) into the heart to regenerate the damaged myocardium [158,159]. However, there is no approved phase III clinical trial for AMI patients, and the results from smaller trials are inconsistent in terms of the efficacy and potential risk of administration routes [158,159]. As such, there is an unmet need to discover effective and safe therapies to promote cardiomyocyte proliferation and heart regeneration. In this section, we focus on potential targets that can modulate cardiac metabolism to promote heart regeneration (summarised in Table 1).

### 4.1. Long Non-Coding RNAs

Long non-coding RNAs (lncRNAs) are non-coding transcripts of >200 nucleotides in length and which are abundantly expressed in the cardiovascular system where they regulate cardiac development and disease [168,169]. LncRNAs are emerging as regulators of glucose and fatty acid metabolism, but only a few cardiac-specific lncRNAs have been investigated to date [170,171]. For instance, the cardiomyocyte-enriched lncRNA, LncHrt, has been shown to preserve cardiac metabolism and improve cardiac function in post-MI adult mice by activating the LKB1–AMPK pathway via sirtuin 2 (Sirt2) [172]. Importantly, lncRNAs have been found to regulate cardiomyocyte proliferation, angiogenesis, and heart regeneration by mediating transcriptional and epigenetic remodelling in the heart [173]. Bioinformatic assessment of neonatal mouse hearts has revealed differentially expressed lncRNAs at P1 and P7 that were associated with cardiac metabolism and cell proliferation [174]. Moreover, overexpression of Sirt1 antisense lncRNA in adult mouse hearts led to the stabilisation of Sirt1 mRNA which promoted an increase in ki67^+^ and PHH3^+^ cardiomyocytes [175]. Accumulating evidence support Sirt1 activity in the positive regulation of PPARα and PGC1α which promotes fatty acid metabolism in phenylephrine-induced cardiomyocytes hypertrophy in neonatal rats [176]. These findings further highlight the controversies regarding the inhibition of FAO to promote cardiomyocyte proliferation [65,66,71].

### 4.2. Hormones

Glucocorticoids are steroid hormones released by adrenal glands in response to stress and play important roles in cardiovascular diseases and in the maturation of foetal cardiac function [177,178,179]. In neonatal mouse hearts, physiological exposure to glucocorticoids was found to reduce cardiomyocyte proliferation, whereas ablation of glucocorticoid receptors extended the time window of cell cycle exit, which coincided with increased expression of glycolytic genes [160]. Consistently, dexamethasone (a potent glucocorticoid) inhibited the proliferation of isolated cardiomyocytes from newborn P2 rats, as evidenced by an increased number of binucleated cardiomyocytes, consisting of reduced cyclin D2 expression [162]. It could be speculated that the mechanism by which glucocorticoids suppress cardiomyocyte proliferation is by modulating FAO, as dexamethasone was shown to induce expression of FAO-related genes in mouse foetal cardiomyocytes [161]. Collectively, these findings suggest that inhibition of glucocorticoid signalling may promote cardiomyocyte proliferation in neonatal mice, but whether this approach can exert similar effects in adult mammalian hearts warrants further investigation; however, caution is advised when attempting to suppress glucocorticoid signalling, as this pathway has been shown to protect cardiomyocytes from cell death [180].

In higher vertebrates, thyroid hormones are secreted by the thyroid gland and exist as two active forms: triiodothyronine (T3) and thyroxine (T4) [181]. The heart is a target organ of thyroid hormones and recent studies have shown this hormone regulates cardiac metabolism through multiple mechanisms [182]. An increase in circulating levels of thyroid hormones have been observed after birth where it triggers cardiomyocyte cell cycle arrest and suppresses regenerative potential [167]. This has led to the speculation that changes in the levels of thyroid hormones could mediate the interplay between metabolism and cell cycle arrest [183]. Consistent with an anti-proliferative role, T3 was found to decrease BrdU uptake in ovine foetal cardiomyocytes by reducing cyclin D1 expression [163]. Similarly, a novel interaction between adrenergic-thyroid hormone in postnatal mice led to an increase in metabolic rates which may impair cardiomyocyte division and limit proliferation [184]. While these findings suggest that reduced thyroid levels promote cardiomyocyte proliferation, it will be challenging to translate this approach to clinical settings as decreased levels of thyroid hormones in the adult heart has been associated with an increased risk of cardiovascular diseases [181]. Conflicting findings have also been reported, whereby T3 administration in neonatal mouse hearts stimulated cardiomyocyte proliferation by inducing mitochondrial ROS production, which, in turn, activated JNK2α2-mediated, IGF1-dependent Erk1/2 proliferative pathways [164]. This finding conflicts with the ROS paradigm where increased ROS production is considered a key suppressor of cardiomyocyte proliferation [22,112,114,143]. In other studies, inhibition of DUSP5 (the nuclear phospho-Erk1/2-specific phosphatase) increased T3-activited phospho-Erk1/2 levels, resulting in ventricular cardiomyocyte proliferation of ~15% in young adult mice [165]. Though T3 has been shown to induce cardiomyocyte proliferation in both neonatal and young adult mice, caution is advised when considering this approach as a therapeutic modality to repair the damaged heart, as high-dose T4 administration has been found to stimulate hypertrophy of existing cardiomyocyte, rather than promote hyperplastic growth [185]. Collectively, these findings suggest that modulation of thyroid hormones can mediate cardiomyocyte proliferation, but it is important to identify the time windows when this hormone should be suppressed or elevated to promote effective regeneration.

To summarise, lncRNAs and hormones are promising targets that could potentially promote cardiomyocyte proliferation via direct and/or indirect modulation of cardiac metabolism. A comprehensive understanding of their mechanistic properties in modulating epigenetics and endogenous influences is key to their further development as therapeutic modalities for repairing the damaged heart in the setting of AMI.

## 5. Conclusions and Future Directions

HF is, in most cases, a progressive condition with a poor prognosis that imposes a global economic burden of ~$108 billion per year. IHD is the most frequent underlying cause of HF, as an ischemic event, can induce substantial cardiomyocyte death which precipitates adverse remodelling and cardiac dysfunction [1,2]. For decades, a vast number of studies have explored the possibility of transplanting cardiac and non-cardiac cells into the damaged myocardium with the aim of restoring cardiac function, either through direct action of the donor cells or via paracrine mechanisms [186]. However, the outcomes of these studies have been controversial, with large clinical studies reporting no obvious improvements in cardiac function following cell transplantation [187]. It would be ideal if we had sufficient knowledge of how to re-activate endogenous pathways that regulate the cell cycle in adult cardiomyocytes, given that the heart is one of the least regenerative organs in the human body [12,13]. Findings from rodents do support the potential for heart regeneration during the early stages after birth [15]; however, the extremely narrow regenerative window in larger mammals [16,17] calls into question whether cardiomyocyte proliferation can be considered a realistic therapeutic option, whilst underscoring the differences in regenerative capacity between species. Future studies in mammals that are more closely related to humans could help to provide novel insight on re-activating the cell cycle in the adult heart.

Changes in cardiac metabolism underlie the pathophysiology of several cardiac diseases [20,30], yet its role in heart regeneration has been barely explored, which is surprising, given that shifts in substrate utilisation before and after birth coincide with the terminal differentiation and reduced proliferation of cardiomyocytes [20,21]. Experimental studies do suggest that reduced oxidative metabolism favours cardiomyocyte re-entry into the cell cycle [137], but this raises the question of why heart regeneration is not initiated in the setting of HF, given that oxidative metabolism is already dampened [188]. Most likely, there are fundamental differences between the metabolism-proliferation axis in foetal and failing hearts, despite both adapting (or maladapting) to a reduced oxidative metabolism profile, and future studies should focus on these differences to elucidate metabolic pathways that can be potentially modulated without predisposing a pathological outcome.

Activation of the renin–angiotensin system (RAS) and increased production of the main effector, angiotensin II (Ang II), are instrumental in cardiac remodelling by promoting myofibroblast proliferation and matrix synthesis. Increased expression of angiotensin-converting enzyme has been observed in cardiomyocytes adjacent to the infarct scar and in nonmyocytes within the scarred tissue in MI patients [189], suggesting that RAS exerts pleiotropic effects on several cell types. Moreover, an increased expression of Ang II receptor type 1 (AT1) has been observed in infarcted rat hearts with increased Ang II binding affinity in the endothelium and myofibers [190]. In cardiomyocytes, Ang II is a potent inducer of hypertrophy, with studies also supporting a pathogenic role in metabolic perturbations. For instance, acute exposure of rat neonatal cardiomyocytes to Ang II was found to result in increased glucose uptake [191], while prolonged exposure of adult rat cardiomyocytes elicited downregulation of FAO pathways [192]. Importantly, these metabolic changes were associated with cardiomyocyte hypertrophy, rather than hyperplasia. RAS playing a role in cardiomyocyte proliferation is unlikely given that its inhibition was not found to mediate cardiomyocyte proliferation post-MI, but did increase vascular densities in the border zone [193]. Consistently, upregulation of AT1 was found to decrease microvessel densities in the setting of MI, while its inhibition promoted angiogenesis [194,195]. Conversely, Ang II can trigger VEGF synthesis in mesenchymal stem cells (MSCs) and injection of Ang II-treated MSCs into the border zone of infarcted hearts led to substantial improvements in cardiac function and reductions in infarct size and fibrosis [196].

Finally, if regeneration were to occur, would the increase in cardiomyocyte (and or endothelial cell) numbers be sufficient to provide meaningful improvements in cardiac function? A typical MI can cause the loss of ~1 billion cardiomyocytes in the adult heart, so the regeneration of this many cardiomyocytes will require a comprehensive understanding of its cell cycle arrest and re-entry pathways. To conclude, the targeting of cardiac metabolism to promote heart regeneration is an attractive concept, but one that comes with many future challenges. Nevertheless, the use of multi-omics platforms coupled with human samples and preclinical models could help to guide this area of research towards identifying suitable targets which can be adopted into clinical practice to improve health outcomes in patients with IHD.

## Figures and Tables

**Table 1:** List of studies investigating the effects of hormones as mediators of cardiomyocyte proliferation and cardiac metabolism.

Hormone	Treatment	Animal/Cellular Model	Effects on Cardiomyocyte Proliferation	Proliferation Markers Evaluated	Effects on Cardiac Metabolism	References
Glucocorticoid	Cardiomyocyte-specific knockout of glucocorticoid receptors	Neonatal C57BL/6 mice (P1)	Promoted	Ki67; BrdU; Aurora B; Nucleation	Decreased fatty acid oxidation; increased glycolysis	[160]
	Dexamethasone	Pregnant C57BL/6J mice (E13.5 or E16.5)			Failed to induce fatty acid oxidation	[161]
		Pregnant C57BL/6J mice (E17.5)			Decreased fatty acid oxidation	
		Neonatal Sprague Dawley rats (P2)	Inhibited	Ki67; Nucleation		[162]
Thyroid hormones	T3	Ovine (*Ovis aries*) foetal cardiomyocytes (~135 days gestation)	Inhibited	BrdU		[163]
		Neonatal cardiomyocytes from C57BL/6 mice (P2-P4)	Increased	PHH3; EdU	Increased ROS production	[164]
		Neonatal C57BL/6 mice (P6)	Increased	PHH3; EdU		[165]
	Thyroidectomy	Neonatal sheep (P30)			Reduced mitochondrial maturation and biogenesis	[166]
	*Myh6-Cre;Thra^DN^* *^/+^*	Neonatal C57BL/6 mice (P14)	Increased	Ki67; EdU; Aurora B; Nucleation	Downregulation of mitochondrial genes	[167]
	*Myh6-Cre;Thra^DN^* *^/+^*	Adult C57BL/6 mice post-IRI injury	Increased	Ki67; EdU; Aurora B; Nucleation		

Abbreviation: *Myh6-Cre;Thra^DN/+^*—cardiomyocyte-specific overexpression of dominant negative thyroid hormone receptor alpha; IRI—ischemia/reperfusion injury; PHH3—phospho-histone H3.
